# The Intersection of Psychological Distress, Cardiovascular Health, and Allostatic Load in Occupational Settings: Implications for Preventive Medicine, Occupational Health, and Clinical Management

**DOI:** 10.3390/medicina62101841

**Published:** 2026-09-23

**Authors:** Eunice Kwan Chae Park, Woo Hyun Park, Eun-Young Jeon

**Affiliations:** 1 Creative Technology Management, Humanities, Arts, and Social Sciences Division, Underwood International College, Yonsei University, Seoul 03722, Republic of Korea; eunicepark@yonsei.ac.kr; 2 Department of Physiology, Medical School, Jeonbuk National University, Jeonju 54896, Republic of Korea; 3 Institute of Humanities, Yonsei University, Seoul 03722, Republic of Korea

**Keywords:** psychological distress, cardiovascular disease, allostatic load, occupational stress, moral distress, systemic inflammation, preventive medicine, public health, clinical management, occupational health

## Abstract

*Background*: The clinical and epidemiological literature establishes a clear link between modern occupational environments and human health, highlighting a significant intersection between psychological distress, ethical dilemmas, and physiological dysregulation. This review evaluates the biological, psychological, and occupational pathways linking workplace distress to Cardiovascular Disease (CVD) through a multidisciplinary preventive medicine lens, while also emphasizing its relevance for occupational health, public health policy, and clinical management. *Methods*: To achieve this, a comprehensive narrative literature search was conducted across PubMed, Scopus, and Web of Science databases to identify relevant peer-reviewed studies published up to August 2026. By utilizing the allostatic load model, this paper explores how chronic activation of the Hypothalamic–Pituitary–Adrenal (HPA) axis and persistent systemic inflammation serve as primary biological mediators bridging psychological exposure and physical disease. Attention is given to how sociodemographic vulnerabilities—including racial and gender disparities—exacerbate these risks. We concurrently examine the lived experiences of patients navigating chronic cardiac disease and the distinct ethical burdens faced by healthcare professionals and medical trainees. *Results*: This synthesis holds significant clinical value by supporting a potential shift from reactive CVD treatments toward comprehensive preventive strategies that address the psychosocial components of physiological wear-and-tear. Specifically, we emphasize targeted health education and cognitive-behavioral interventions integrated within comprehensive clinical and organizational frameworks. By elucidating the molecular mechanisms, broader clinical realities, and structural implications of occupational stress, this comprehensive review informs proactive clinical and occupational interventions. *Conclusions*: Ultimately, addressing psychological distress within the workplace is essential for mitigating the global burden of cardiovascular morbidity. This approach narrows persistent health disparities and advances the integration of psychology and public health into modern clinical practice.

## 1. Introduction

The biological and behavioral mechanisms linking psychological distress to excess morbidity and mortality remain a key area of investigation. This spans occupational health, medical sociology, and cardiovascular medicine. This suggests the utility of an integrated approach between psychological sciences and preventive medicine. Growing awareness exists within the medical community. However, a synthetic gap remains. Researchers have not fully synthesized how specific occupational hazards translate into measurable cardiovascular pathology. These hazards range from the physical demands of manual labor to the moral distress experienced by healthcare professionals and medical trainees. The primary objective of this investigation is to synthesize the molecular mechanisms of allostatic load, identify how socio-occupational environments relate to health inequalities, and critically evaluate contemporary therapeutic and occupational interventions. By systematically mapping these biological pathways and evaluating structural disadvantages, this synthesis provides an evidence-based foundation for multidisciplinary healthcare teams, clinical specialists, occupational health practitioners, and policymakers. This foundation helps implement targeted preventive measures that safeguard cardiovascular health, mitigate health inequalities, and promote effective stress-management strategies across clinical and workplace frameworks.

Psychological distress was historically viewed as a secondary symptom, but it is increasingly recognized as a contributing factor to Cardiovascular Disease (CVD) and a significant complication of the disease. This bidirectional relationship is evident in the clinical reality of survivorship. A substantial proportion of patients experience post-Myocardial Infarction (MI) psychological distress, which is associated with a notable increase in future cardiac events. This burden operates through both behavioral pathways, such as reduced physical activity and decreased medication adherence, as well as direct physiological inflammatory mechanisms [1]. Furthermore, large-scale multi-cohort studies have noted psychological distress as a strong, independent predictor of both all-cause and cancer-specific mortality. The underlying biological bridge is primarily systemic inflammation, which explains a considerable portion of this elevated mortality risk. This substantiates the link between psychological well-being and mortality, demonstrating that these risks persist independently of general lifestyle factors like diet or body mass [2].

Researchers rely on the concept of allostatic load to contextualize how psychological distress alters the human body over time. Allostatic load is clinically characterized as a maladaptive biological process that occurs when physiological stability—allostasis—is compromised due to repeated and chronic stress exposure. Over time, this negatively affects both physical and cognitive function, accelerating cellular aging. Consequently, this theoretical model is highly suited for understanding the long-term health effects of occupational stress. In these settings, individuals are exposed to high cognitive, emotional, and physical demands [3]. This impact of stress on the cardiovascular system extends across the lifespan, ranging from tracking neurodevelopmental outcomes in individuals born with Congenital Heart Disease (ConHD) [4] to analyzing the psychological fatigue of frontline workers and medical students navigating high-stakes learning environments. The intersection of psychological distress, structural disadvantage, and cardiovascular health is evident in the modern workforce. High-strain occupational and clinical environments contribute notably to systemic inflammation and neuroendocrine dysfunction. Because these environments frequently involve ethical dilemmas that compound the baseline cognitive dissonance experienced by workers and learners, there is a critical need for multidisciplinary health interventions and structured occupational support systems in professional training.

## 2. Literature Search Strategy and Methodology

To synthesize the current evidence, a comprehensive narrative literature search was performed using the PubMed, Scopus, and Web of Science databases, encompassing the peer-reviewed literature published from inception up to August 2026. The search strategy utilized combinations of the following keywords: “psychological distress,” “allostatic load,” “occupational stress,” “moral distress,” “systemic inflammation,” and “cardiovascular disease.” Selection criteria prioritized articles published in English, focusing on large-scale epidemiological cohorts, rigorous clinical trials, and foundational pathophysiological mechanisms. A narrative review design was intentionally selected over a systematic quantitative analysis to allow for a broader, multidisciplinary conceptual synthesis that spans molecular biology, medical sociology, and preventive clinical interventions.

## 3. Biological Mechanisms: Allostasis, Inflammation, and the HPA Axis

### 3.1. The Allostatic Load Model in Occupational and Clinical Health

The physiological translation of psychological distress into CVD is primarily mediated by the neuroendocrine and immune systems. The allostatic load model captures the cumulative physiological wear-and-tear on these systems, particularly focusing on the Hypothalamic–Pituitary–Adrenal (HPA) axis. This wear-and-tear results from chronic over- or underactivity of systems that normally promote adaptation. The transition from acute, adaptive stress responses to chronic, maladaptive allostatic load is heavily influenced by the environment. Specifically, the stability of the work or clinical environment plays a major role. For instance, longitudinal research demonstrates the effects of major workplace reorganizations. Rigorous academic transitions, such as entering medical residency, also show similar effects. These events trigger measurable increases in allostatic load, indicating that corporate and institutional instability alters employee and student physiology [5]. Figure 1 outlines the conceptual pathway from psychological triggers to physiological outcomes, illustrating how occupational exposure translates to systemic disease. To further quantify this biological wear-and-tear, Table 1 provides a summary of the primary physiological biomarkers associated with this systemic damage.

This alteration extends to occupational syndromes such as burnout and cynicism. These syndromes are related to higher allostatic load, independent of age, sex, education, and occupation. Burnout is strongly associated with elevated allostatic load, although co-occurring depression explains a substantial portion of this specific association. This overlap emphasizes the potential benefit of dual clinical management. Occupational burnout and psychiatric distress should be managed together within employee health services and professional wellness programs [13]. Importantly, the chronicity of these stressors influences the biological response. Experience sampling studies with healthcare professionals provide insight into this process. Daily negative work events predict psychological distress responses. However, they do not always trigger acute salivary cortisol spikes. This suggests that the persistent accumulation of stress primarily drives systemic allostatic load [14].

The neuroendocrine system may experience significant dysregulation in cases of persistent occupational trauma. Researchers evaluating victims of workplace mobbing have documented a paradoxical hypoactivity of the HPA axis. This blunted cortisol response underscores a critical danger. Chronic work-related psychological distress can eventually impair the neuroendocrine system’s capacity to mount a normal stress response [10]. The COVID-19 pandemic further highlighted this phenomenon. Broad population studies confirmed that persistent anxiety, depression, and abnormal illness behaviors became strongly associated with elevated allostatic load across medical and nonmedical workers [15]. This indicates a notable gap in psychological preparedness. This gap warrants attention in future preventive health strategies and clinical resource planning.

### 3.2. Systemic Inflammation and Biomarkers

While neuroendocrine pathways initiate the stress response, systemic inflammation serves as the primary effector of cardiovascular damage. Inflammation is often viewed as a consequence of disease. However, clinical studies consistently demonstrate that inflammatory activation frequently precedes and sustains psychological distress. Prospective studies of white-collar employees demonstrate that baseline shifts in inflammatory mediators predict subsequent increases in psychological distress and depression. Crucially, these inflammatory shifts exhibit distinct sex differences. Specific variations in cytokines like Interferon-gamma (IFN-γ) and Tumor Necrosis Factor-alpha (TNF-α) predict distress differently in men and women, demanding nuanced clinical interpretation [7].

Biomarkers such as Interleukin-6 (IL-6) play a dynamic, predictive role in this inflammatory cascade. Elevated IL-6 levels are strongly associated with the development of psychological distress. Conversely, the resolution of depressive symptoms over time is reliably predicted by lower baseline IL-6 levels. Recovering individuals show corresponding decreases in systemic IL-6 [6]. Beyond transient cytokines, Heat Shock Proteins—particularly Heat Shock Protein 60 (Hsp60)—serve as critical markers linking stress to CHD susceptibility. In cohorts of civil servants, elevated plasma Hsp60 was positively associated with pro-inflammatory cytokines and correlated negatively with markers of endothelial health. Furthermore, Hsp60 levels demonstrated a significant association with psychosocial vulnerabilities, such as low Socioeconomic Status (SES), social isolation, and heightened psychological distress [9].

When these occupational or environmental stressors reach extreme levels, this inflammatory link becomes clearly evident. Individuals exposed to severe conflict and terrorism experience much higher levels of Post-Traumatic Stress Disorder (PTSD). This significantly correlates with elevated C-Reactive Protein (CRP) and heightened viral antibody levels. This firmly establishes the biological embedding of severe psychological trauma. It also demonstrates its direct translation into cardiovascular vulnerability [16].

### 3.3. Epigenetic and Long-Term Consequences

The reach of this biological embedding is not confined to the individual’s immediate career span. It can begin as early as the prenatal period and carry through to adulthood via epigenetic modifications. Prospective pilot studies tracking mother-offspring pairs demonstrate the impact of maternal perceived stress during pregnancy. This stress is associated with long-term alterations in the DNA methylation patterns of the glucocorticoid receptor gene promoter in adult offspring. Because this gene is crucial for receptor function, these methylation changes may profoundly influence the offspring’s long-term HPA-axis regulation, rather than acting as an absolute determinant. This suggests that early-life exposure to psychological distress predisposes individuals to heightened cardiovascular and psychological vulnerability decades later. It renders stress a truly transgenerational health determinant [12].

## 4. Occupational Stress and Cardiovascular Outcomes Across Professions

The burden of allostatic load is influenced by the specific nature of an individual’s occupation. Work environments with high demands, low predictability, and physical hazards create acute and chronic cardiovascular risks. Cognitive dissonance further compounds these risks. The biological mechanisms remain consistent. However, their professional manifestations vary significantly. This suggests the need for context-specific educational and preventive strategies across different sectors. Table 2 provides a breakdown of how specific work environments impact physiological health. It analyzes the moral and ethical stressors faced by healthcare professionals within high-acuity settings. Additionally, Figure 2 provides a comparative model. It illustrates how distinct occupational stressors manifest clinically across different sectors.

### 4.1. Transport, Manual Labor, and Industrial Roles

Data from demanding manual and transport professions highlight the severity of occupational cardiovascular risk. The sedentary, high-vigilance nature of certain jobs yields extreme physiological costs. For instance, studies of train drivers reveal concerning health statistics, with a majority exhibiting obesity, hypertension, and dyslipidemia. Ultimately, this occupation was independently associated with a drastically increased likelihood of having cardiometabolic issues and psychological distress compared to control groups [24].

This physical–psychological nexus is also apparent in manual roles such as forklift truck operation. Physical and psychological demands intersect here to produce measurable biological changes. Operators report significantly higher levels of anxiety alongside increased rates of work-limiting physical pain. Demonstrating the physiological depth of this strain, multivariate data analysis showed that a specific profile of plasma inflammatory biomarkers could accurately discriminate these operators from healthy subjects. This highlights the intricate relationship between biomechanical strain, psychological distress, and systemic inflammation [25]. Expanding to the broader industrial workforce, low predictability at work acts as a severe independent risk factor for acute MI. Prospective evidence demonstrated that a chronic lack of work predictability substantially increases the long-term risk of acute MI. This effect is heavily driven by middle-aged employees facing peak financial and familial responsibilities [26].

### 4.2. Science, Technology, and Corporate Environments

Moving from the tangible strain of manual labor to professional and academic sectors, the drivers of allostasis change. Prolonged cognitive demands, job insecurity, and unrecognized burnout replace physical strain. Among female scientists and technicians, severe burnout and overwhelming stress are directly linked to a much higher CVD risk. A notable percentage of women in these high-pressure fields reported early cardiovascular issues and depressive symptoms. This points to a bidirectional relationship exacerbated by occupational insomnia and chronic stress [27].

Beyond the burden of high-pressure output, the anticipation of job loss during corporate downsizing significantly elevates psychological distress. During large-scale layoffs, the vast majority of affected employees reported high anxiety. Employment in targeted departments was associated with clinically significant increases in blood pressure, independent of baseline BMI and lifestyle factors [28]. The drive to maintain employment during such instability often leads to presenteeism (working while ill). Acute presenteeism contributes more to the slow, insidious accumulation of allostatic load than acting as a sudden ischemic trigger for coronary events [29].

When these occupational stressors act upon employees already suffering from cardiometabolic diseases, the results often prove difficult to overcome. Modifiable risk factors like obesity and psychological distress strongly predict permanent work disability and early labor market exit. Individuals with pre-existing heart or cerebrovascular disease face a markedly higher risk of disability pension, with psychological distress driving a substantial portion of this premature exit [30]. Finally, occupational hazards extend beyond traditional psychological stress to physical environmental exposures. These exposures cause severe secondary psychological distress. For instance, subjects diagnosed with occupational asthma often suffer permanent impairment and a severe decline in quality of life. The prevalence of clinical depression and anxiety is remarkably high due to sudden livelihood loss and the exhaustion of managing chronic symptoms [31].

## 5. Moral Distress, Cognitive Dissonance, and Ethical Dilemmas in Healthcare Environments and Practice

Industrial and corporate roles face pressures of predictability and insecurity. In contrast, healthcare professionals and medical students represent a uniquely vulnerable population. They face a frequent intersection of high workload, emotional labor, and ethical dilemmas. Moral distress occurs when professionals know the ethically appropriate action to take. However, they are constrained from acting upon it by internal, organizational, or external barriers. As outlined in Figure 2, these environmental pressures map onto distinct cardiovascular outcomes within the healthcare sector. This highlights the importance of integrating ethics and resilience training early in medical practice and nursing protocols.

### 5.1. The Intensive Care Unit Experience

The Intensive Care Unit (ICU) serves as a focal point for moral distress and subsequent psychological morbidity. Evaluations of ICU doctors and nurses during the COVID-19 pandemic revealed substantial psychological strain. The vast majority of staff experienced moderate to high levels of stress, and a significant portion exhibited symptoms of PTSD. Nurses experienced more severe symptoms of psychological distress compared to physicians. This was driven by perceived physical and emotional safety threats, enforced role changes, and societal stigma. The absence of robust organizational support further exacerbated the situation [17]. This distress was compounded by shifts in routine care. Physicians frequently missed clinical round opportunities for non-COVID-19 conditions. This led to a heavy reliance on nursing staff. It also added to the moral distress of clinicians unable to provide their standard level of care [32]. This highlights the value of incorporating crisis-management and emotional resilience training into standard nursing guidelines and residency programs.

Even outside of pandemic conditions, the baseline psychological toll on Cardiovascular Surgery Intensive Care Unit (CVICU) professionals is high. It demonstrates that moral distress and burnout are closely related. Non-physician professionals, particularly Registered Nurses and Respiratory Therapists, consistently exhibit higher moral distress scores than physicians. High levels of burnout syndrome are widely prevalent in these settings. The core of this burden involves significant cognitive dissonance regarding end-of-life care. Clinicians struggle with witnessing other healthcare providers giving unrealistic expectations to grieving families [18]. Addressing this challenge requires expanding beyond clinical skills training. It suggests the need to integrate structured didactic modules on palliative communication and ethical conflict resolution in allied health practice.

### 5.2. Ethical Dilemmas in Treatment and Patient Consent

Clinical decision-making frequently forces healthcare providers to balance patient autonomy against non-maleficence beyond the ICU. For example, prescribing complex psychiatric medications carries risks of cardiac complications. Balancing psychiatric stability against cardiac risk causes psychological distress for the care team. It requires complex risk–benefit analyses without clear guidelines [19].

Administering acute therapies without explicit consent under emergency exceptions introduces legal and moral anxiety. This is common in stroke therapy due to narrow treatment windows and impaired patient capacity [20]. Similarly, critical patients sometimes explicitly refuse life-saving cardiovascular interventions. Respecting this refusal challenges the clinician’s duty to preserve life and generates moral distress [21]. This ethical balance is deeply ingrained in the medical profession’s history. Early clinical research experiments required researchers to weigh scientific progress against patient endangerment [33]. Today, debates surrounding prophylactic antibiotic use in neurosurgery highlight similar tensions. These debates raise questions of professional equipoise and informed consent [34]. Routine situations also present ethical clashes. For example, navigating regulations for home occupational therapy creates conflicts between employer expectations and a therapist’s moral values [35]. Specialized settings introduce unique challenges. In aviation medicine, flight surgeons must balance pilot medical confidentiality against public safety mandates, which adds to their psychological burden [36]. The management of brain death represents another trigger for this psychological burden. Families sometimes request continued mechanical ventilation after Death by Neurological Criteria (DNC) is declared. Clinicians then face a conflict between compassionate accommodation and professional integrity. Indefinite demands often force stressful clinical actions. These actions can permanently fracture the provider-family relationship [22]. Emergency scenarios also present complex decisions regarding futile interventions in highly vulnerable elderly patients. Significant delays in seeking care correlate directly with higher mortality. This exacerbates the surgical team’s moral distress when operating under challenging conditions [23]. These compounding ethical challenges suggest a need for proactive organizational support. Current clinical guidelines and training programs could benefit from a heavier emphasis on navigating moral ambiguity. They should also focus on understanding the physiological toll of clinical decision-making.

### 5.3. Palliative Care and Chronic Cardiovascular Management

To mitigate these ethical crises, integrating palliative care into advanced CVD management is crucial. It alleviates systemic distress for both patients and providers. Palliative care focuses on communication, shared decision-making, and advance care planning. It provides comprehensive alleviation of physical, emotional, and psychological suffering. However, structural barriers and a historical focus on highly technical interventions frequently delay palliative consultations in cardiology. This systemic delay prolongs patient suffering and increases provider burnout. Consequently, professional societies strongly suggest that palliative care be integrated much earlier in the disease trajectory for advanced heart disease. This requires robust health professional education and significant policy shifts for reimbursement [37,38]. Even during the height of the COVID-19 pandemic, the appropriate use of limited clinical resources engendered agonizing ethical decisions. This proves that modern healthcare requires the active incorporation of measures designed specifically to assuage moral distress and burnout in psychologically exhausted staff [39].

## 6. Demographic Vulnerabilities: Intersecting Risks of Race, Gender, and SES

While occupational hazards provide the spark for allostatic load, demographic factors determine the sensitivity of the fuel. The impact of occupational stress, psychological distress, and allostatic load on cardiovascular health is heavily moderated by demographic factors, creating distinct vulnerability profiles that require targeted epidemiological interventions. For a comprehensive overview of how these disparities manifest in clinical outcomes and the statistical realities backing them, please refer to Table 3, which explicitly maps these sociodemographic vulnerabilities to specific cardiovascular risk profiles.

### 6.1. Racial and Ethnic Disparities

Racial minorities frequently carry a much higher allostatic load. This is due to the synergistic effects of occupational stress, socioeconomic disadvantage, and systemic discrimination. Research on Black young adults has demonstrated a direct link between racial stress, psychological distress, and cardiovascular risk indicators. Internalized racism has a profound effect on metabolic markers, while individual experiences of racial stress are associated with elevated blood pressure. Furthermore, clinical depression in these cohorts is significantly associated with vascular strain, suggesting that the relationship between racial stress and early-onset CVD requires urgent clinical attention [40]. This risk profile persists into adulthood, deeply exacerbated by occupational factors such as job insecurity, unemployment, and low perceived job control [44].

These vulnerabilities are further shaped by cultural and environmental factors. In the UK, South Asian populations experience disproportionately high rates of CHD compared to other ethnic groups. An assessment of physical activity levels revealed consistently lower metabolic activity scores in these populations. This substantial ethnic group difference remained robust and independent of self-reported health, SES, and psychological distress. This highlights that underlying behavioral and cultural factors must also be comprehensively addressed alongside psychological distress [41].

### 6.2. Gender Disparities

Gender significantly modulates both the experience of psychological distress and its ultimate cardiovascular manifestations. Women consistently report higher levels of psychological distress in response to chronic illness and occupational stressors. In patients surviving In-Hospital Cardiac Arrest, gender plays a massive role in recovery trajectories. Female survivors report significantly worse overall health status, higher clinical anxiety, and more severe symptoms of depression compared to men. This indicates a critical need for individualized, gender-specific psychosocial support [42].

In the workplace, women’s cardiovascular and mental health are uniquely impacted by specific organizational expectations. Occupational burnout and psychological distress in women are strongly associated with difficulties in reconciling private life and work life. Lack of recognition and the persistent fear of losing one’s job are also major drivers. Conversely, for male workers, distress is often tied more directly to immediate time pressure and lack of physical resources [43]. Furthermore, psychological distress and social alienation increase hypertension incidence more prominently in women than in men. This occurs chiefly through a strong secondary association with health risk behaviors [44]. However, the objective biological manifestation of this distress can differ. While baseline psychological distress increases the incidence of self-reported CHD in both genders, specific ECG abnormalities are often linked to psychological distress primarily in men, showing nuanced gender differences in biological disease manifestation [45].

### 6.3. Socioeconomic Status and Age Trajectories

The most pervasive demographic modifier remains socioeconomic status. Across all demographics, low SES acts synergistically with psychological distress to amplify cardiovascular mortality. People in lower socioeconomic circumstances are significantly more vulnerable to the adverse cardiovascular effects of psychological distress. The mortality risk related to increased psychological distress is highly significant in low-SES individuals, while often remaining non-significant in highly affluent groups. This demonstrates that financial stability actively buffers the heart against stress [46]. Figure 3 visually demonstrates the synergistic effects of low socioeconomic status, racial stress, and gender disparities on cardiovascular mortality.

This embedding is a lifelong process. Intergenerational income stratification plays a substantial mediating role in adult health outcomes. Adult income mediates almost all of the relationship between childhood poverty and midlife self-rated health and psychological distress. This cements the role of lifelong financial security in establishing biological cardiovascular resilience [47]. As these populations age, the trajectory intersects critically with cardiometabolic factors. Physical frailty and cardiometabolic decline in the elderly are intimately linked with severe psychological distress, acting as profound markers for steeply increased suicide risk [48]. This culminates in a self-perpetuating cycle where poor physical health perpetuates lifelong psychological suffering [49]. Alarmingly, this cycle may begin in youth. Metabolic Syndrome is increasingly prevalent among obese youth in rapidly modernizing regions, with subjective psychological distress found in the vast majority of these subjects [50].

## 7. The Bidirectional Path: Patient Psychological Distress and Cardiovascular Pathology

The preceding sections discussed how occupational and demographic stress relates to CVD. However, the diagnosis itself acts as a novel stressor. The bidirectional relationship between CVD and psychological distress is clinically relevant. Distress precipitates CVD through chronic allostatic pathways. It also emerges as an adverse consequence of cardiac diagnoses. This subsequent distress impairs rehabilitation, medical adherence, and quality of life. This bidirectional relationship highlights the need for comprehensive educational methodologies. Medical students and practitioners must be trained to address the psychosocial dimensions of disease alongside biological symptoms.

### 7.1. The Psychological Burden of Chronic Illness

A CVD diagnosis universally elevates the risk for clinical depression, anxiety, and PTSD. National health surveys estimate that psychological distress prevalence is significantly higher in patients with heart failure or prior myocardial infarction compared to non-diseased populations. Despite strong associations between distress and adverse cardiac events, only a small fraction of distressed cardiac patients consult mental health professionals. This highlights a substantial systemic care gap [51]. Furthermore, heart attack and stroke survivors experience significantly higher anxiety and depression, making them much more likely to suffer serious psychological distress [52]. The necessity of a holistic recovery model was concurrently emphasized during the post-COVID-19 era. Mitigating malnutrition and systemic inflammation was vital for patients facing compounding psychological distress and fatigue [53].

Beyond objective prevalence, the individual’s cognitive processing heavily influences their illness adaptation. Heart failure patients often accurately attribute their illness to psychosocial factors but dangerously underestimate clinical risks like hypertension. This causal misunderstanding correlates with poorer health and paradoxically less threatening illness views, directly hindering recovery [54]. Consequently, post-MI distress is frequently a stronger predictor of failed social adjustment than the organic disease’s severity. Because psychiatric-level distress affects a vast number of post-MI patients, anxiety reduction remains a non-negotiable rehabilitation target [55]. This suffering is further compounded by cognitive decline following cardiac arrest. Self-reported cognitive deficits correlate strongly with poorer health, increased anxiety, and depression [56]. Finally, this psychological burden extends outward to caregivers. Stroke caregiver burden is deeply exacerbated by low family support and external stressors. Caregivers historically exhibit alarming anxiety, depression, and hostility, underscoring a critical need for caregiver-focused interventions [57,58].

### 7.2. Arrhythmias, Implanted Devices, and Diagnostic Distress

Unpredictable cardiac conditions and implanted electronic devices introduce unique, persistent stressors. In adults with ConHD, experiencing atrial arrhythmias is associated with significantly worse physical health, psychological distress, and illness perception [59]. Similarly, patients with current or past heart failure report severely diminished physical and mental functioning [60]. Thoracic Aortic Disease also causes marked quality of life impairments, particularly in younger females, largely driven by fears of aortic rupture [61]. Even successful surgical recovery induces extreme distress; patients recovering from vascular interventions often undergo a strenuous recovery where unmanaged physical discomfort generates severe anxiety [62].

A particularly acute stressor is the Implantable Cardioverter-Defibrillator (ICD). While life-saving, ICDs often induce a threatening illness perspective. Recipients with secondary prevention ICDs exhibit lower quality of life than primary prevention cohorts, as the constant anticipation of traumatic shocks creates a hyperaroused state driving allostatic load [63]. Conversely, mitigating this fear through brief cognitive behavioral preimplantation programs shows substantial success. Home-based intervention plans significantly improve physical health, radically reduce clinical anxiety, and lower unplanned hospital readmissions [64].

The psychological weight of cardiovascular care begins as early as routine diagnostics. While cardiovascular screening in athletes generally fosters safety, true-positive detections frequently trigger acute distress, necessitating accessible psychological resources prior to screening [65]. Similarly, genetic assessments for inherited CVD produce intense uncertainty, requiring multidisciplinary models integrating clinical psychologists [66]. Predictive DNA testing for late-onset disorders reveals complex mechanisms: high pre-test distress may represent effective mental preparation, whereas abnormally low distress might indicate dangerous denial-avoidance behavior [67]. Finally, communication gaps exacerbate conditions like venous thromboembolism in vulnerable cancer patients. Professionals often avoid discussing risks to spare patients emotional burden, yet this prevents patients from recognizing life-threatening symptoms. This represents a failure to balance psychological comfort with clinical safety [68].

### 7.3. Specialized Cohorts and Comorbidities

The impact of psychological distress impairs physical recovery and adherence across numerous medical contexts. While distress is evident in non-cardiac conditions such as gynecological cancers [69] and diabetes [70], these comorbidities uniquely compound the burden on the cardiovascular system. Insomnia in cancer survivors is multifactorial—involving systemic inflammation and HPA axis dysregulation—and significantly reduces therapy adherence [71]. This distress extends to family members, advocating for routine professional psychosocial support [72].

Furthermore, self-employed individuals managing cardiac diseases face heightened psychological and financial distress. Work limitations are directly associated with demoralization, heavily mediated by the perceived difficulty of maintaining business operations while ill [73] Intolerance of uncertainty further exacerbates this relationship, necessitating targeted occupational support [74]. Methodological challenges arise when patients’ psychological coping mechanisms confound research. Stressful life events are sometimes negatively associated with blood pressure, yet positively associated with somatic complaints and anxiety. This suggests that repressive coping mechanisms obscure simple stress-disease models, highlighting the critical need to account for reporting biases [75]. Finally, in severe pulmonary hypertension, depression and anxiety are prevalent yet undertreated. Patients show high rates of depressive symptoms, highlighting an urgent need for targeted behavioral interventions [76]. Consequently, managing these psychological factors must become standard care across cardiorespiratory diseases [77]. Crucially, the intense psychological distress and poor adherence exhibited by these multimorbid patient populations directly contribute to the emotional labor and moral distress that healthcare professionals must navigate daily, thereby firmly bridging patient outcomes with occupational health.

### 7.4. The Pediatric and Parental Psychological Burden

Diagnosing a child with ConHD or a complex genetic syndrome inflicts profound distress on parents. This subsequently impacts the child’s clinical outcomes. For parents caring for individuals with complex syndromes, distress is exacerbated by inadequate welfare support systems and educational mismatches [78]. Parents endure decades of distress stemming from societal stigma. However, phenomenological studies indicate this persistent hardship paradoxically fosters deep psychological growth when adequate emotional support is eventually provided [79]. Mothers facing heart surgery for young children experience intense role pressure and severe psychological distress [80]. Similarly, fathers describe profound suffering and require intense family support, demonstrating that paternal distress mirrors maternal intensity following a sudden diagnosis [81].

While peer support offers parents deep emotional relief, it requires professional moderation. Online surveys reveal that exposing parents to traumatic experiences of other children can trigger severe secondary psychological distress and withdrawal [82]. The complexity deepens for parents raising children with concurrent ConHD and mental health issues. Parents frequently perceive a direct connection between the physical and psychological conditions. This emphasizes the need for integrated pediatric psycho-cardiology care to prevent delays in psychiatric support [83]. Surveys highlight a substantial hidden burden on caregivers, with many reporting severe anxiety and seeking extensive sick leave. This signals an unmet need for dedicated community care services [84]. Finally, prenatal ConHD diagnoses demand robust counseling. Pregnant women and partners emphasize the necessity of clear medical information and pediatric liaison nurses to navigate severe prenatal distress and complex ethical decisions [85]. Navigating the profound suffering of these families and managing complex ethical decisions places a heavy psychosocial burden on pediatric healthcare providers. This highlights the critical intersection between severe patient/family distress and occupational burnout within pediatric clinical settings.

## 8. Preventive Strategies, Occupational Health Interventions, and Clinical Therapeutics

Medical paradigms are gradually shifting toward multimodal interventions due to the evidence of a mind-heart link. Psychological distress, allostatic load, and CVD form an interwoven web. Addressing this web requires evidence-based interventions. These interventions must target both the individual’s psychological resilience and the broader occupational or clinical environment. These concepts should be integrated into preventive medicine protocols, occupational health guidelines, and multidisciplinary clinical practice. This integration may better equip future professionals with necessary coping strategies. Table 4 provides a synthesized comparison of these modalities. Furthermore, these targeted treatments may modulate HPA-axis dysregulation and improve clinical outcomes. Figure 4 delineates these multimodal intervention pathways.

### 8.1. Psychological and Behavioral Therapies in Practice and Prevention

Biological and psychological feedback loops can be addressed through targeted psychotherapies. Acceptance and Commitment Therapy (ACT) delivered in structured group settings decreases systemic inflammatory markers. Clinical trials examined working-age adults with psychological distress. Face-to-face ACT generated physiological improvements in these individuals compared to control groups. This suggests that enhancing psychological flexibility yields measurable benefits on systemic inflammation [8]. Building on this, group-based ACT protocols are currently deployed for older stroke survivors. These protocols target experiential avoidance and post-stroke depression [89]. Such therapeutic frameworks offer valuable models for clinical training. They could be implemented as routine supportive measures within psychiatric and cardiovascular care programs.

Cognitive Behavioral Therapy (CBT) also demonstrates efficacy in cardiac populations. Brief CBT interventions for defibrillator patients reduce psychological morbidity. They also enhance physical capacity and lower hospital readmissions [64]. To optimize these therapies, tools like the Cardiac Distress Inventory (CDI) are currently undergoing psychometric validation to help professionals identify specific psychosocial concerns post-cardiac event [90]. A key tenet of modern intervention is recognizing varied psychological distress phenotypes. This recognition allows for tailored treatment pathways. For instance, pain coping skills training can be matched to specific distress phenotypes. This yields better clinical outcomes for patients affected by catastrophizing and kinesiophobia [91]. Similarly, validated patient-reported outcome measures allow clinicians to isolate disease-specific distress from general depression [92]. The success of these programs requires the integration of allied health professionals. Surveys show that speech-and-language therapists recognize the psychosocial impact of chronic conditions. However, they frequently lack confidence in addressing psychological needs. This identifies a potential gap in current allied health practice. It suggests the utility of integrated psychological protocols within their clinical routines [93]. Furthermore, collaborative methodologies are pioneering personalized interventions for rare diseases [94]. These approaches utilize virtual focus groups and narrative interviews.

Therapeutic efficacy is also modulated by personality traits. These traits influence surgical recovery. Following coronary artery bypass graft surgery, the Type D personality acts as a vulnerability factor for poorer outcomes. Anxiety largely mediates the influence of Type D personality on deterioration in health-related quality of life. This emphasizes that underlying distress should be addressed in postoperative routines [95]. Recovery is further complicated by specific distress syndromes like alexithymia. This is prevalent in systemic lupus erythematosus patients and necessitates comprehensive multidisciplinary care [96]. Furthermore, physical impairments post-MI are linked to psychological states. Psychological impairment correlates with physical functioning in cardiac rehabilitation programs. Therefore, physical therapists should address psychological barriers alongside physical ones [97].

However, engaging certain demographic groups in these interventions remains difficult. Studies offering targeted CBT to men with poor emotional adjustment post-MI found minimal interest. This indicates a need for novel, less stigmatizing methods to involve male patients [98]. Addressing these demographic and cultural barriers should become a core competency within medical practice. Clinicians must learn how to design and communicate psychological interventions that successfully overcome gender-specific stigmas and encourage patient participation.

### 8.2. Workplace Stress Reduction and Mindfulness

Clinical therapies address the individual, while workplace interventions target the occupational environment. Tailored psychological interventions are proving effective in lowering allostatic load in high-stress occupations. Mindfulness training programs for law enforcement led to a lower cortisol awakening response. They also led to measurable improvements in sleep quality. This indicates that mindfulness provides neuroendocrine buffering against occupational trauma [11]. Integrating evidence-based mindfulness or stress-reduction principles into medical and nursing protocols has shown promise in mitigating trainee burnout. Furthermore, workplace stress reduction programs have yielded physiological benefits in corporate environments. These programs focus on positive emotion refocusing and emotional restructuring. Employees participating in these programs exhibited clinically significant reductions in blood pressure post-intervention [86]. While psychological conditioning is important, specific nutritional regimens touted for recovery must be carefully evaluated. Studies of professional athletes showed that specific dietary supplementation did not significantly affect inflammatory cytokine responses. This emphasizes that recovery from intense strain requires comprehensive rest rather than reliance on isolated supplements [99].

### 8.3. eHealth, Rehabilitation, and Collaborative Care

The digital era introduces scalable solutions for managing cardiovascular distress. Systematic reviews demonstrate that internet-based CBT alleviates depressive symptoms in CVD patients. It offers an accessible adjunct to traditional rehabilitation [88]. These digital therapies may effectively mitigate HPA-axis dysregulation as part of the broader therapeutic trajectory (Figure 4). Comprehensive cardiac rehabilitation is equally critical. Outpatients completing structured phase II programs post-MI exhibit lower job stress and psychological distress. This helps prevent psychosocial deterioration upon returning to work [87]. Similarly, structured home-based programs improve recovery confidence and reduce anxiety better than passive information booklets. This indicates the importance of active rehabilitation models [100].

Recent clinical trials have further expanded the therapeutic frontier. The ESCAPE trial protocol outlines a patient-centered BCC pathway utilizing care managers and eHealth platforms aimed at improving HRQOL in multimorbid heart failure patients [101]. Moreover, machine learning is being employed to identify patients at high risk for depressive symptoms post-surgery. This utilizes psychometric and inflammatory variables to inform automated screening and early preventive strategies [102]. However, the successful implementation of these interventions requires standardized objective measurements. Widely used capacity tools often lack psychometric validation in specific survivor populations [103]. Furthermore, multidisciplinary teams optimize medical management and lower psychological distress among treating physicians by sharing complex decision-making burdens [104]. Physical evaluation techniques are also evolving. They reveal that psychological factors account for substantial variance in tissue remodeling over time [105].

The long-term success of lifestyle modification depends on understanding psychological barriers beyond technological tools. Psychological distress and poor self-control hinder physical activity in CHD patients. This necessitates customized rehabilitation plans [106]. However, current delivery of risk factor modification is often inadequate. Healthcare professionals frequently lack confidence in managing psychological distress in cardiac patients [107]. This highlights the need for advanced communication protocols within clinical practice. Enhancing direct patient communication can lower patient confusion and resistance [108]. Patient confusion correlates with elevated psychological distress and poor physiological control. This emphasizes the clinical importance of trust-building communication [109]. Integrating narrative medicine further enhances outcomes. Multimodal programs that validate the patient’s lived story reduce pain and improve functioning compared to strictly biomedical approaches [110].

Systemic factors and existential support play a decisive role alongside clinician–patient interactions. Patient education is critical for correcting misconceptions about surgical risk. Risk perception often illogically correlates with expected benefit. This demands improvements in preoperative communication to alleviate distress [111]. Furthermore, trained spiritual professionals can play a role in acute care. Intense religious struggle is associated with anxiety, depression, and sleep disturbances among hospitalized patients [112]. At the macro level, system performance directly impacts these outcomes. Robust patient rights and clear information access are associated with better perceived physical health and lower health risk behaviors. This proves that transparent health infrastructures inherently lower patient psychological burden [113]. Addressing these dimensions at a national policy level is necessary as metabolic diseases increase [114]. Finally, the complexity of these interventions must be contextualized through gender disparities. Higher decision authority at work is specifically associated with a lower obesity risk in women [115]. Outside formal settings, patients frequently turn to online peers for medical sense-making. Without accessible professional education, they become vulnerable to unproven approaches that paradoxically exacerbate distress [116].

## 9. Limitations and Future Directions

The current literature provides evidence linking psychological distress to cardiovascular pathology via allostatic load. However, several limitations within the existing research must be acknowledged. A primary limitation is the reliance on cross-sectional study designs. This restricts the ability to draw definitive causal inferences between psychological distress and specific cardiovascular endpoints. Longitudinal data capturing the dynamic interplay of inflammatory markers and psychological states over time remain sparse. Furthermore, the narrative review design chosen for this study inherently limits systematic reproducibility and is susceptible to selection bias, presenting a methodological constraint compared to systematic reviews. This design was prioritized to permit a broad, multidisciplinary conceptual synthesis, but future systematic evaluations are certainly warranted. Furthermore, there is a lack of validated psychometric and physiological assessment tools tailored to niche medical subpopulations. This limits the generalizability of certain findings. Additionally, psychological intervention studies frequently struggle with demographic selection biases. This indicates that current intervention models may fail to engage certain demographics due to stigma or program design.

Looking ahead, future directions for research and clinical implementation should proactively address these limitations. The medical field is exploring advanced predictive modeling and machine learning to predict psychological deterioration. Prospective studies are utilizing clinical, psychometric, and inflammation-related variables. These variables algorithmically predict depressive symptoms and PTSD following major cardiac surgery. Concurrently, future research should evaluate long-term clinical and occupational outcomes. Rigorous methodologies should determine the efficacy of embedding these psychological interventions directly into standard medical care and workplace wellness programs. These sophisticated models and preventive frameworks may facilitate earlier screening and psychiatric intervention for high-risk individuals. This can occur before allostatic load causes irreversible damage. Ultimately, this preserves human capital, supports employee well-being, and sustains workforce participation. Finally, occupational health policies may benefit from shifting focus. The focus should move from treating individual employee burnout to implementing systemic organizational reforms. Corporate and clinical policies should also be adapted. They must address the underlying factors of low work predictability, moral distress in healthcare, and institutional cognitive dissonance. This contributes to a more resilient workforce.

## 10. Conclusions

In summary, psychological distress, ethical dilemmas, and occupational burnout are not merely transient emotional states. The current literature supports the concept that they act as biological variables. They drive allostatic load and manifest in established CVD and systemic pathology. Consequently, they impact long-term psychological well-being. The chronic activation of the HPA axis and the elevation of systemic inflammatory markers (IL-6, CRP, and TNF-α) play a crucial role. They serve as a physiological bridge between psychological state and cardiovascular health.

Healthcare professionals and medical students bear a substantial, yet frequently unrecognized, burden of moral distress. This burden degrades job satisfaction and creates psychological morbidity. Recognizing that the impact of these stressors is moderated by race, gender, SES, and age is important. It is essential for developing equitable public health and preventive strategies. Healthcare systems, policymakers, clinical leaders, and employers face a new imperative. They should move beyond merely treating the downstream physiological symptoms of CVD. Several steps can address this issue. We can integrate targeted psychological therapies and improve healthcare communication. We can also integrate psycho-educational training programs within formal medical practice and foster supportive, predictable occupational environments. Through these actions, it may be possible to lower allostatic load and alleviate psychological distress. This would ultimately mitigate the global burden of stress-induced cardiovascular pathology. While acknowledging the methodological limitations inherent in narrative syntheses, advancing the integration of health, psychology, and preventive medicine in modern medical practice represents a vital step. It is necessary for ensuring the long-term sustainability and well-being of the healthcare workforce.

## Figures and Tables

**Figure 1:**
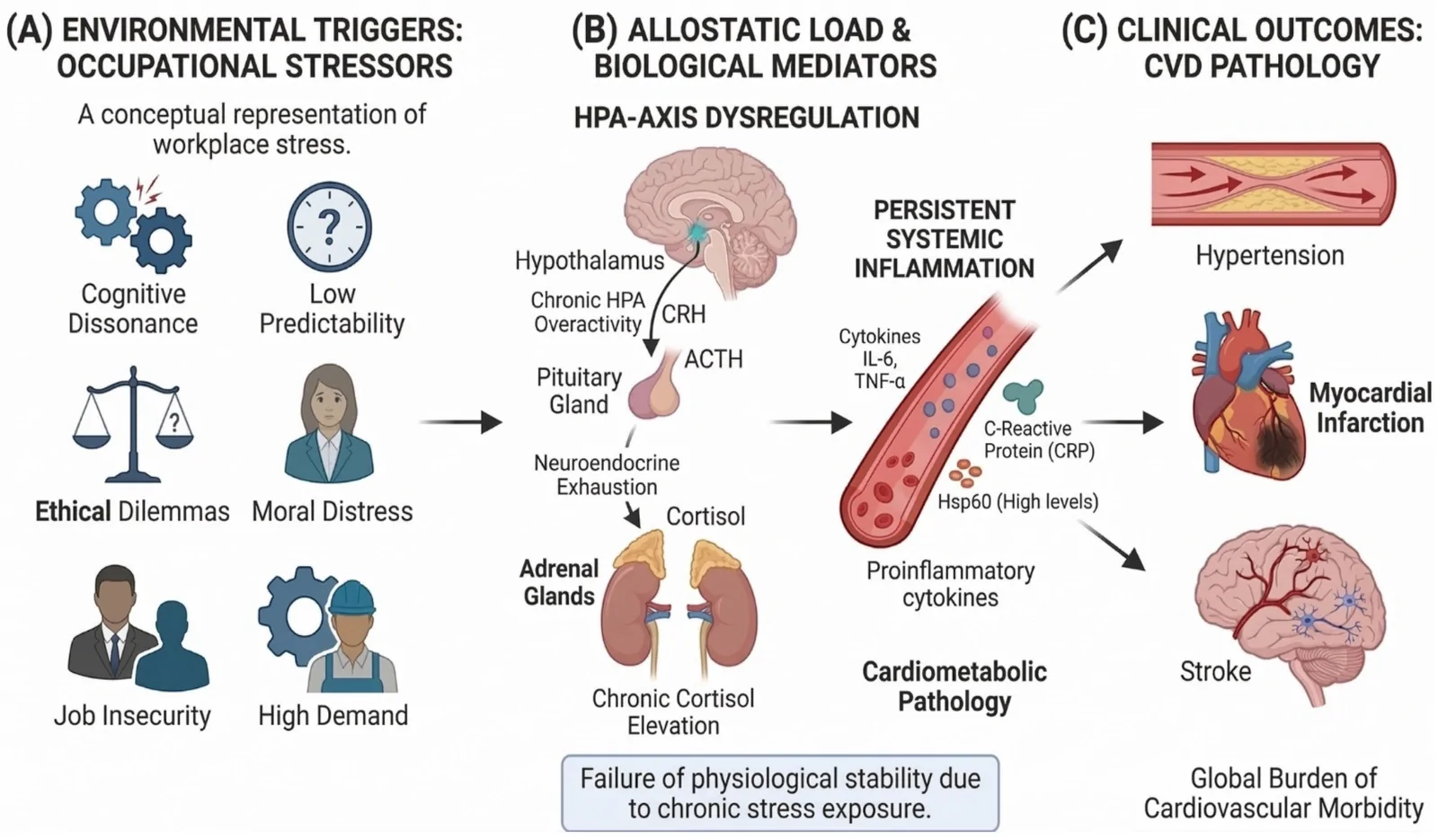
Conceptual framework illustrating the biological trajectory from occupational stress to cardiovascular disease (CVD) pathology. The model outlines a sequential cascade divided into three primary domains: (**A**) Environmental Triggers: Represents key occupational stressors in the workplace, including cognitive dissonance, low predictability, ethical dilemmas, moral distress, job insecurity, and high demand. (**B**) Allostatic Load and Biological Mediators: Demonstrates HPA-axis dysregulation driven by chronic stress. Continuous exposure leads to chronic HPA overactivity and subsequent neuroendocrine exhaustion, altering the signaling cascade of CRH, ACTH, and cortisol released from the adrenal glands. (**C**) Clinical Outcomes: Illustrates how neuroendocrine dysfunction promotes persistent systemic inflammation, marked by elevated proinflammatory cytokines (IL-6, TNF-α), C-reactive protein (CRP), and high levels of Hsp60. This underlying cardiometabolic pathology ultimately manifests as severe clinical outcomes, including hypertension, myocardial infarction, and stroke, directly contributing to the global burden of cardiovascular morbidity. The fundamental mechanism driving this entire pathway is the failure of physiological stability due to chronic stress exposure.

**Figure 2:**
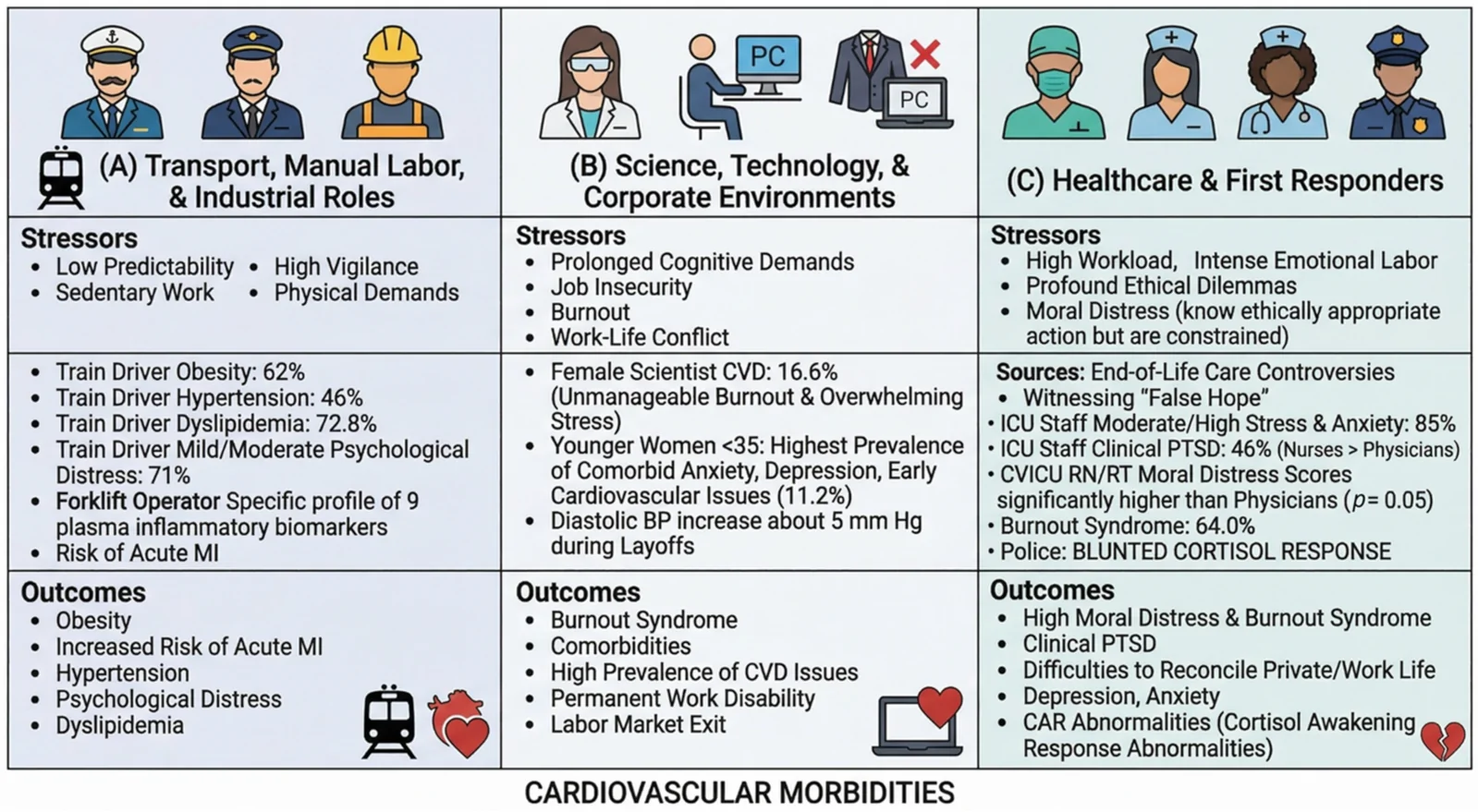
Professional and clinical manifestations of occupational stress across diverse work environments leading to distinct cardiovascular morbidities. This conceptual diagram visualizes how occupational stress presents clinically across different sectors, acting through varying mechanisms to produce distinct cardiovascular morbidities. The model is structured across three primary cohorts, detailing specific stressors and resulting pathologies: (**A**) Transport, Manual Labor, and Industrial Roles: This section focuses on physical and vigilance-related stressors (e.g., in train drivers and forklift operators) such as low predictability, high vigilance, sedentary work, and physical demands. These environmental triggers correlate with high prevalent cardiometabolic risk factors and specific inflammatory biomarker profiles, ultimately leading to direct cardiometabolic pathologies, including obesity, hypertension, dyslipidemia, and an increased risk of acute myocardial infarction (MI), concurrently with psychological distress. (**B**) Science, Technology, and Corporate Environments: This panel highlights cognitive and job security concerns (e.g., in female scientists and young professional women), including prolonged cognitive demands, job insecurity, burnout, and work–life conflict. Evidence indicates that these pressures, exacerbated by unmanageable burnout, lead to early cardiovascular issues and an elevated physiological response during instability. The resulting outcomes are characterized by a profound psychosocial burden, comorbidities, permanent work disability, and early labor market exit. (**C**) Healthcare and First Responders: This column emphasizes intense emotional labor and ethical stressors (e.g., in ICU staff, CVICU RN/RT, and police), such as high workload, profound ethical dilemmas, and moral distress. The biological cascade is evidenced by specific HPA axis dysregulation and severe psychological outcomes, including high rates of stress, anxiety, and clinical PTSD. The resulting morbidities constitute a unique cluster of high psychological burden, CAR abnormalities, and functional impairment, such as burnout and difficulties reconciling private/work life.

**Figure 3:**
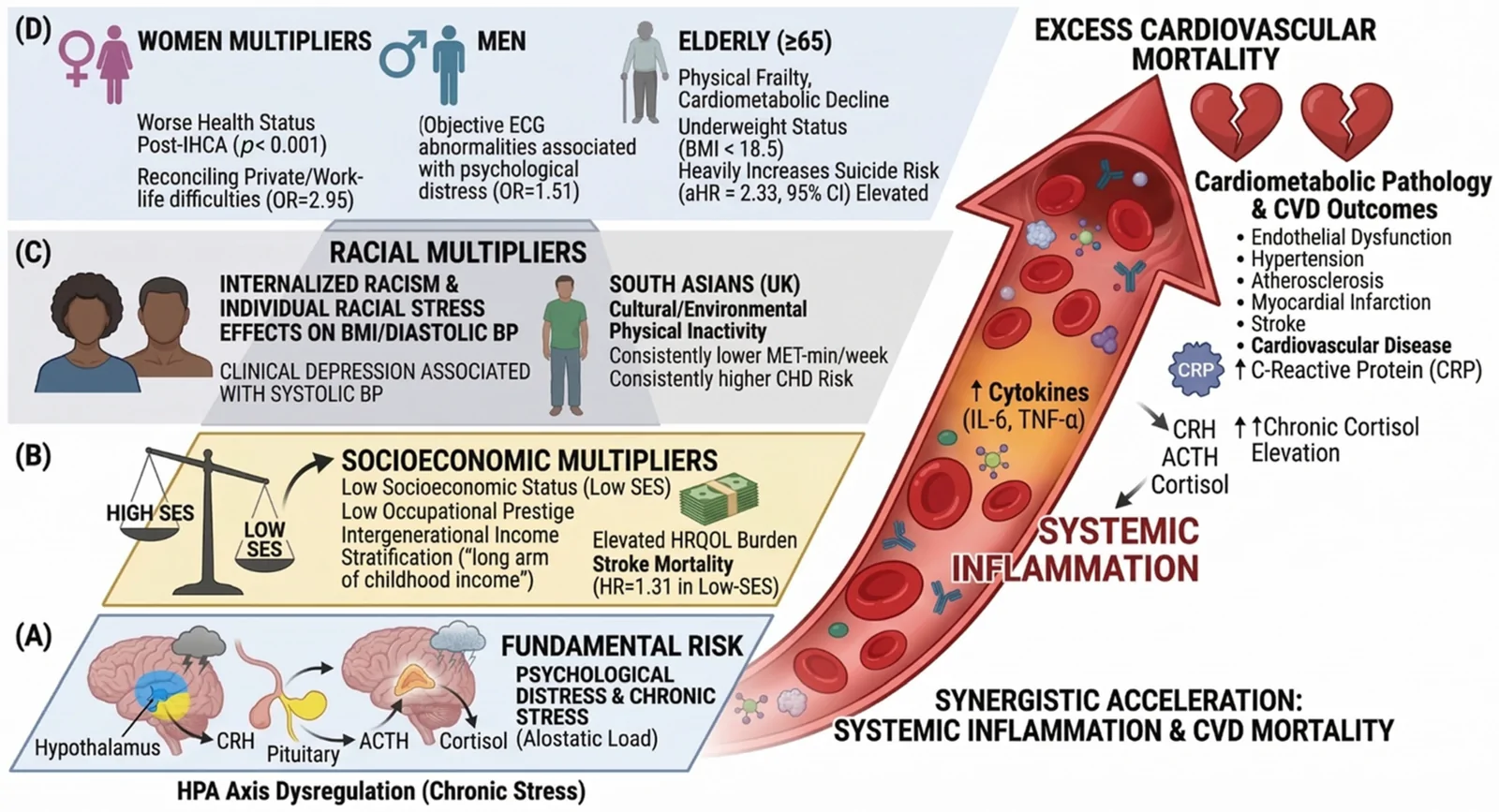
Conceptual framework illustrating the multi-level determinants and biological trajectory of cardiovascular disease (CVD) mortality, emphasizing intersecting sociodemographic vulnerabilities. The model integrates a synergistic cascade divided into distinct domains: (**A**) Fundamental Risk: Chronic psychological distress drives HPA-axis dysregulation via the CRH-ACTH-cortisol signaling cascade, leading to chronic cortisol elevation and allostatic load. (**B**–**D**) Intersecting Sociodemographic Multipliers: Specific variables amplify the biological effects of distress: (**B**) Socioeconomic factors (e.g., low SES, low occupational prestige, intergenerational income stratification) elevate HRQOL burden and stroke mortality (HR = 1.31). (**C**) Racial factors demonstrate how internalized racism and racial stress impact BMI and blood pressure, while cultural/environmental physical inactivity (e.g., in UK South Asians) consistently increases CHD risk. (**D**) Demographic factors highlight gender-specific risks—such as worse post-IHCA health, work–life conflicts, and distress related to work–life balance (OR = 2.95) in women, and stress-related ECG abnormalities (OR = 1.51) in men—alongside geriatric vulnerabilities (≥65 years) like physical frailty and underweight-associated suicide risk (aHR = 2.33). Systemic Inflammation and Clinical Outcomes: The synergistic acceleration of these fundamental risks and sociodemographic multipliers promotes persistent systemic inflammation (elevated IL-6, TNF-α, and CRP). This underlying pathway ultimately manifests as irreversible cardiometabolic pathology (e.g., endothelial dysfunction, atherosclerosis, myocardial infarction, and stroke), driving excess cardiovascular mortality.

**Figure 4:**
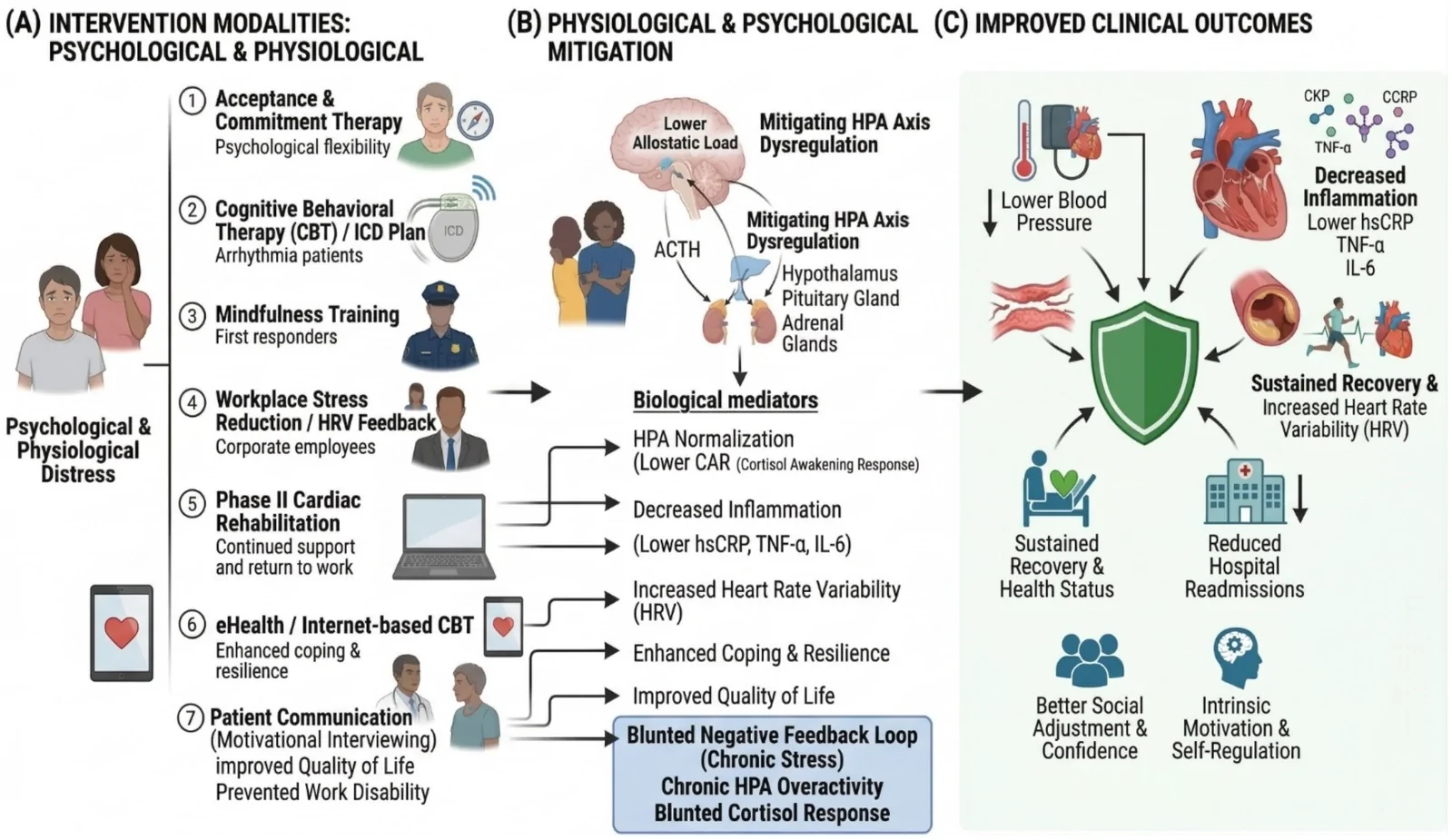
Multimodal intervention pathways mitigating allostatic load and improving cardiovascular outcomes. This conceptual model delineates the therapeutic trajectory from targeted treatments to clinical recovery across three primary domains: (**A**) Intervention Modalities: Highlights a comprehensive spectrum of psychological and physiological strategies—ranging from Acceptance and Commitment Therapy (ACT) and Cognitive Behavioral Therapy (CBT) to workplace stress reduction, eHealth platforms, and motivational interviewing—utilized to address baseline distress. (**B**) Physiological and Psychological Mitigation: Illustrates the mechanistic impact of these interventions in reversing HPA-axis dysregulation and lowering allostatic load. Key mediating processes include HPA normalization (e.g., correcting the blunted negative feedback loop and lowering the Cortisol Awakening Response [CAR]), increasing Heart Rate Variability (HRV), and reducing systemic inflammation (hsCRP, TNF-α, IL-6), coupled with enhanced cognitive resilience. (**C**) Improved Clinical Outcomes: Demonstrates the ultimate protective benefits (visualized by the central shield) of these biological and psychological shifts, culminating in lower blood pressure, sustained cardiovascular recovery, reduced hospital readmissions, and enhanced patient self-regulation and social adjustment.

**Table 1:** Primary Biomarkers of Allostatic Load and Systemic Inflammation. This table summarizes the key neuroendocrine and immune markers utilized in the current literature to quantify physiological wear-and-tear resulting from chronic psychological distress, trauma, and occupational burnout. It highlights their specific biological systems, clinical significance in disease progression, and the key associated studies defining their predictive power for cardiovascular events and mortality.

Biomarker	Biological System	Study Design/Sample Size	Clinical Significance and Observation	Statistical/Physiological Impact	Key References
IL-6	Immune/Inflammatory	Cohort study (Non-clinical sample with psychological distress)	Predicts symptom resolution in psychological distress; elevated levels are associated with distress, while recovery is marked by a decrease in IL-6.	Low baseline IL-6 predicts symptom resolution (age/sex-adjusted RR = 1.15, 95% CI 1.06–1.25).	[6]
TNF-α	Immune/Inflammatory	16-Month Prospective Study (*n* = 104; 61 men, 43 women)	Inflammatory activation precedes distress. Higher TNF-α levels predict subsequent increases in psychological distress (K6 scores) specifically in men.	Serves as a direct predictor of future psychological distress before clinical symptoms emerge.	[7]
IFN-γ	Immune/Inflammatory	16-Month Prospective Study (*n* = 104; 61 men, 43 women)	Exhibits distinct sex differences: lower IFN-γ predicts distress in men, whereas higher IFN-γ predicts depressive symptoms in women.	Demonstrates that systemic inflammation pathways leading to depression are highly gender-specific.	[7]
hsCRP	Immune/Inflammatory	RCT (*n* = 204 working-age adults)	Face-to-face ACT specifically targets and reduces hsCRP in distressed, overweight adults.	Significant group × time interaction (*p* = 0.012) showing physiological reduction in inflammation via psychotherapy.	[8]
Hsp60	Cellular Stress/Immune	Whitehall II Cohort (*n* = 229 British civil servants)	Positively associated with TNF-α and negatively with von Willebrand factor. Elevated levels linked to low SES, social isolation, and distress in women.	~20% of participants had >1000 ng/mL, a concentration likely to induce adverse biological vascular effects.	[9]
CAR	Neuroendocrine (HPA axis)	RCT (*n* = 114 police officers); Cross-sectional (mobbing victims)	Blunted in victims of severe chronic mobbing; CAR is significantly lowered by 8-week mindfulness training.	Reflects either HPA axis exhaustion in chronic trauma or physiological recovery post-mindfulness intervention.	[10,11]
NR3C1 Methylation	Epigenetic	Prospective Pilot Study (*n* = 52 mother-offspring pairs)	Maternal perceived stress during pregnancy alters NR3C1 promoter methylation in offspring well into adulthood (ages 28–29).	Absolute methylation levels changed significantly (−1.62% to +5.89%, *p* < 0.05), calibrating lifelong HPA axis regulation.	[12]

Note: ACT: Acceptance and Commitment Therapy, CAR: Cortisol Awakening Response, CI: Confidence Interval, HPA axis: Hypothalamic–Pituitary–Adrenal axis, hsCRP: High-Sensitivity C-Reactive Protein, Hsp60: Heat Shock Protein 60, IFN-γ: Interferon-gamma, IL-6: Interleukin-6, NR3C1: Nuclear Receptor Subfamily 3 Group C Member 1, RCT: Randomized Controlled Trial, RR: Risk Ratio, SES: Socioeconomic Status, TNF-α: Tumor Necrosis Factor-alpha.

**Table 2:** Sources of Moral Distress and Ethical Dilemmas Among Healthcare Professionals. This comprehensive table categorizes specific clinical scenarios that generate severe cognitive dissonance and burnout in medical staff. Scenarios include end-of-life care controversies in ICUs, administration of thrombolytics without explicit consent in acute stroke, prescribing cardiotoxic psychiatric medications, and managing the extension of physiological support after brain death. It outlines the dilemma description, the resulting psychological impact on staff, and the key associated literature.

Clinical Scenario/Specialty	Description of Ethical Dilemma	Resulting Psychological Impact on Professionals	Statistical/Clinical Evidence	Key References
ICU/COVID-19	Enforced role changes, perceived safety threats, and navigating severe social stigmatism while managing resource scarcity.	Severe stress, anxiety, and clinical PTSD, particularly in nursing staff.	85% experienced moderate/high stress; 46% exhibited symptoms of PTSD.	[17]
CVICU	Controversies over end-of-life care (e.g., maintaining hopeless patients on ventilators) and witnessing providers give “false hope” to families.	High moral distress and burnout syndrome; negative perception of job satisfaction and emotional exhaustion.	64.0% of respondents showed high burnout; non-physicians (RN/RT) had significantly higher distress scores (*p* = 0.05).	[18]
Psychiatry/Cardiology	Prescribing clozapine for treatment-resistant schizophrenia causing life-threatening pericarditis.	Cognitive dissonance balancing psychiatric stability against severe, potentially fatal physical cardiac risk.	Forces complex risk–benefit analyses that lack clear clinical guidelines, resulting in profound provider anxiety.	[19]
Emergency Medicine/Acute Stroke	Administering thrombolytics without explicit consent in narrow time windows; treating critical patients who refuse care.	Legal and moral anxiety regarding patient autonomy versus the physician’s fundamental duty to preserve life.	Confounding management decisions that frequently lead to acute moral distress and fear of legal repercussions.	[20,21]
Neurology/Critical Care	Families requesting the extension of physiologic support (ventilation) after DNC has been declared.	Conflict between compassionate family accommodation and professional integrity/stewardship of scarce resources.	“Finite-goal accommodation” mitigates distress, but indefinite demands force definitive, highly stressful clinical actions.	[22]
Vascular Surgery	Rupture of known AAA in elderly patients previously deemed inoperable due to comorbidities.	Agonizing decisions regarding futile interventions versus the imperative to act in acute crises.	Overall mortality of 56%; patient delays in seeking care averaged 20.4 h, directly correlating with operative death (*p* = 0.04).	[23]

Note: AAA: Abdominal Aortic Aneurysm, CVICU: Cardiovascular Surgery Intensive Care Unit, DNC: Death by Neurological Criteria, ICU: Intensive Care Unit, PTSD: Post-Traumatic Stress Disorder, RN: Registered Nurse, RT: Respiratory Therapist.

**Table 3:** Cardiovascular Risk Profiles Stratified by Sociodemographic Vulnerabilities (Race, Gender, SES, and Age). This table delineates how variables such as internalized racism in minority youth, female-specific work–life conflicts and unique physiological stress presentations, intergenerational poverty/low SES, and geriatric physical frailty amplify the biological impact of psychological distress. It explicitly maps demographic factors to specific cardiovascular morbidities and the proposed mechanisms driving these disparities as established by major epidemiological cohorts.

Demographic Factor	Specific Vulnerability/Exposure	Associated Cardiovascular and Health Outcomes	Statistical/Epidemiological Evidence	Key References
Race (Black Young Adults)	Internalized racism and individual racial stress.	Elevated BMI and heightened diastolic BP; clinical depression associated with systolic BP.	Significant effect of internalized racism on BMI; significant effect of racial stress on diastolic BP in 124 subjects.	[40]
Race (South Asians in the UK)	Culturally or environmentally driven physical inactivity.	Extremely low physical activity acts as an independent risk factor for high rates of CHD.	Total MET-min/week consistently lower (973 vs. 1465, *p* < 0.001) than white participants, independent of SES or distress.	[41]
Gender (Women)	Difficulties reconciling private/work life; low occupational status; post-cardiac arrest recovery.	Significantly worse health status post-IHCA; low-status work independently predicts incident hypertension.	Post-IHCA women report significantly higher anxiety and depression (*p* < 0.001); OR = 2.95 for distress related to work–life balance.	[42,43,44]
Gender (Men)	Psychological distress manifesting as objective ECG abnormalities.	Increased incidence of self-reported CHD and clinically observable ECG abnormalities driven by distress.	OR = 1.51 (95% CI 1.1–2.1) for ECG abnormalities in men; not consistently demonstrated in women.	[45]
SES (Low SES)	Low occupational prestige; intergenerational income stratification.	Acts as a profound synergistic multiplier for stress-induced cardiovascular death and stroke.	HR = 1.31 for stroke mortality and HR = 1.33 for mortality from stroke/CHD when combined with psychological distress (compared to high SES).	[46,47]
Age (Elderly ≥ 65)	Physical frailty, cardiometabolic decline, underweight status.	Intimately linked with severe psychological distress, culminating in fatal mental health outcomes (suicide).	Underweight (BMI < 18.5) heavily increases suicide risk (aHR = 2.33, 95% CI 1.20–4.52); higher waist circumference lowers risk (aHR = 0.60).	[48]

Note: aHR: Adjusted Hazard Ratio, BMI: Body Mass Index, BP: Blood Pressure, CHD: Coronary Heart Disease, CI: Confidence Interval, ECG: Electrocardiographic, HR: Hazard Ratio, IHCA: In-Hospital Cardiac Arrest, MET: Metabolic Equivalents of Task, OR: Odds Ratio, SES: Socioeconomic Status.

**Table 4:** Efficacy of Clinical and Occupational Interventions on Psychological and Physiological Distress. This table provides a detailed comparative analysis of modern therapeutic approaches. It covers interventions such as ACT for systemic inflammation, CBT and the “ICD Plan” for arrhythmia patients, and mindfulness training for first responders. It highlights the target populations, key physiological/psychological outcomes, and the seminal associated studies.

Intervention Type	Target Population	Study Design	Key Physiological/Psychological Outcomes	Statistical/Clinical Significance	Key References
ACT	Working-age adults with psychological distress and overweight/obesity.	RCT (*n* = 204)	Face-to-face ACT group sessions target psychological flexibility and successfully reduce systemic inflammation.	Significant decrease in hsCRP at 36-week follow-up (*p* = 0.012 interaction).	[8]
CBT—“ICD Plan”	Patients receiving ICDs.	Multicentered RCT (*n* = 192)	Improved physical health, decreased incidence of clinical anxiety and depression, and vastly reduced hospital readmissions.	50% reduction in unplanned hospital readmissions (11% vs. 22%, *p* < 0.01); higher physical health scores (*p* < 0.01).	[64]
Mindfulness Training	First responders (Police officers).	RCT (*n* = 114)	Provides long-term buffering against chronic occupational stress, improving sleep and lowering physiological arousal.	Significantly lower CAR; sustained improvements at 3-month follow-up.	[11]
Workplace Stress Reduction	Hypertensive corporate employees.	RCT with 3-month follow-up (*n* = 38)	Positive emotion refocusing and HRV feedback drastically lower resting BP and global distress.	Mean adjusted reduction of 10.6 mm Hg (systolic) and 6.3 mm Hg (diastolic); significant reductions in depression.	[86]
Phase II Cardiac Rehabilitation	Middle-aged patients returning to work post-MI.	Prospective cohort (*n* = 109)	Continued structured support actively prevents chronic psychosocial collapse upon returning to the workforce.	Significantly lower job stress, reduced psychological distress, and higher SF-36 HRQOL scores at 6 months.	[87]
eHealth/Internet-based CBT	Patients with varied CVDs.	Meta-Analysis of 12 RCTs (*n* = 2319)	Highly scalable, accessible adjunct to traditional cardiac rehabilitation for patients unable to attend in-person clinics.	Significantly alleviated depressive symptoms (SMD = −0.30, 95% CI −0.47 to −0.14, *p* < 0.001).	[88]

Note: ACT: Acceptance and Commitment Therapy, BP: Blood Pressure, CAR: Cortisol Awakening Response, CBT: Cognitive Behavioral Therapy, CI: Confidence Interval, CVD: Cardiovascular Disease, HRQOL: Health-Related Quality of Life, HRV: Heart Rate Variability, hsCRP: High-Sensitivity C-Reactive Protein, ICD: Implantable Cardioverter-Defibrillator, MI: Myocardial Infarction, RCT: Randomized Controlled Trial, SF-36: 36-Item Short-Form Health Survey, SMD: Standardized Mean Difference.

## Data Availability

No new data were created or analyzed in this study. Data sharing is not applicable to this article.

## Abbreviations

22q11DS: 22q11.2 Deletion Syndrome, 6MWT: 6-Minute Walk Test, AAA: Abdominal Aortic Aneurysm, ACT: Acceptance and Commitment Therapy, aHR: Adjusted Hazard Ratio, AYA: Adolescent and Young Adult, BCC: Blended Collaborative Care, BMI: Body Mass Index, BP: Blood Pressure, CABG: Coronary Artery Bypass Graft, CAR: Cortisol Awakening Response, CBT: Cognitive Behavioral Therapy, CDI: Cardiac Distress Inventory, CEA: Carotid Endarterectomy, CHD: Coronary Heart Disease, CHF: Congestive Heart Failure, CI: Confidence Interval, ConHD: Congenital Heart Disease, CRP: C-Reactive Protein, CTEPH: Chronic Thromboembolic Pulmonary Hypertension, CVD: Cardiovascular Disease, CVICU: Cardiovascular Surgery Intensive Care Unit, DD: Diabetes Distress, DNC: Death by Neurological Criteria, DSD: Disease-Specific Distress, ECG: Electrocardiographic, HPA: Hypothalamic–Pituitary–Adrenal, HR: Hazard Ratio, HRQOL: Health-Related Quality of Life, HRV: Heart Rate Variability, hsCRP: High-Sensitivity C-Reactive Protein, Hsp60: Heat Shock Protein 60, ICD: Implantable Cardioverter-Defibrillator, ICU: Intensive Care Unit, IFN-γ: Interferon-gamma, IHCA: In-Hospital Cardiac Arrest, IL-6: Interleukin-6, IU: Intolerance of Uncertainty, LRFM: Lifestyle and Risk Factor Modification, MCEQ: Medical Consultation Experience Questionnaire, MET: Metabolic Equivalents of Task, MI: Myocardial Infarction, MS: Metabolic Syndrome, NHIS: National Health Interview Survey, NR3C1: Nuclear Receptor Subfamily 3 Group C Member 1, OPLS-DA: Orthogonal Partial Least Square-Discriminant Analysis, OR: Odds Ratio, PAH: Pulmonary Arterial Hypertension, PCI: Percutaneous Coronary Intervention, PE: Pulmonary Embolism, PH: Pulmonary Hypertension, PPS: Preparticipation Screening, PROM: Patient-Reported Outcome Measure, PSD: Post-Stroke Depression, PTSD: Post-Traumatic Stress Disorder, RADS: Rheumatoid Arthritis Distress Scale, RCT: Randomized Controlled Trial, RN: Registered Nurse, RR: Risk Ratio, RT: Respiratory Therapist, RUSI: Rehabilitative Ultrasound Imaging, SES: Socioeconomic Status, SF-36: 36-Item Short-Form Health Survey, SLE: Systemic Lupus Erythematosus, SMD: Standardized Mean Difference, TAD: Thoracic Aortic Disease, TNF-α: Tumor Necrosis Factor-alpha, VTE: Venous Thromboembolism.
